# Supplementary material for: DCLK1 Drives EGFR-TKI-Acquired Resistance in Lung Adenocarcinoma by Remodeling the Epithelial–Mesenchymal Transition Status
Source: Biomedicines. 2023 May 22;11(5):1490. doi: 10.3390/biomedicines11051490 (PMC10216632; doi:10.3390/biomedicines11051490)
Supplement: Supplementary file 1 [file biomedicines-11-01490-s001.zip › biomedicines-2233898-supplementary.pdf]

Table S1. All primers sequences.

Primers names	Assay	Primers sequences (5'→3')
Human-E-cadherin-Forward	RT-PCR	GTCACTGACACCAACGATAATCCT
Human-E-cadherin-Reverse	RT-PCR	TTTCAGTGTGGTGATTACGACGTTA
Human-N-cadherin-Forward	RT-PCR	TCAGGCGTCTGTAGAGGCTT
Human-N-cadherin-Reverse	RT-PCR	ATGCACATCCTTCGATAAGACTG
Human-ZO1-Forward	RT-PCR	CAACATACAGTGACGCTTCACA
Human-ZO1-Reverse	RT-PCR	CACTATTGACGTTTCCCCACTC
Human-ZEB1-Forward	RT-PCR	GATGATGAATGCGAGTCAGATGC
Human-ZEB1-Reverse	RT-PCR	ACAGCAGTGTCTTGTTGTTGT
Human-Vimentin-Forward	RT-PCR	CCTGAACCTGAGGGAAACTAA
Human-Vimentin-Reverse	RT-PCR	GCAGAAAGGCACTTGAAAGC
Human-Snail-Forward	RT-PCR	GCCATGTCCGGACCCACACTG
Human-Snail-Reverse	RT-PCR	GGCAGGGGCAGGTATGGAGA
Human-GAPDH-Forward	RT-PCR	GGAGCGAGATCCCTCCAAAAT
Human- GAPDH -Reverse	RT-PCR	GGCTGTTGTCATACTTCTCATGG
